# Stereotactic body radiotherapy for early glottic cancers: Is this “The Way”?

**DOI:** 10.1016/j.ctro.2025.100962

**Published:** 2025-04-13

**Authors:** Jennifer Le Guévelou, Audrey Larnaudie, Pierre Blanchard, Yoann Pointreau, Joël Castelli

**Affiliations:** a Laboratoire de traitement de l’image et du signal, University of Rennes, France; b Department of Research, Centre Eugène Marquis, Rennes, France; c Department of Radiotherapy, Centre François Baclesse, Caen, France; d Department of Radiotherapy, Institut Gustave Roussy, Université Paris Saclay, Villejuif, France; e Department of Radiotherapy, Institut inter-régional de Cancérologie (ILC) – Centre jean Bernard, Centre de cancérologie de la Sarthe (CCS) – Clinique Victor Hugo, Le Mans, France; f Department of Radiotherapy, Centre Eugène Marquis, Rennes, France

**Keywords:** Stereotactic body radiotherapy, Early glottic cancer, Toxicity

## Abstract

In the past decades, several options have been designed in order to preserve vocal function in patients with early glottic cancer, such as transoral laser microsurgery, new surgical strategies such as partial laryngectomy and radiation therapy (RT). With the development of new radiation techniques enabling a more accurate delivery of radiation dose, and the possibility to deliver high dose per fraction while sparing adjacent organs-at-risks (OARs), stereotactic body radiotherapy (SBRT) is being increasingly tested in this disease setting. We aimed to shed light on this appealing strategy, as well as to underline both optimal target population and specific technical considerations.

## Highlights

• Patient with limited tumor extent and no active smoking represent the ideal target population to receive SBRT.
• SBRT performed in an unselected population can result in severe laryngeal toxicity.
• SBRT for early glottic cancer needs to be prospectively compared to current standard treatment modalities.

## Introduction

With the development of new radiation techniques enabling a more accurate delivery of radiation dose, and the possibility to deliver high dose per fraction while sparing adjacent organs-at-risks (OARs), stereotactic body radiotherapy (SBRT) became a standard of care for various tumor sites including lung and prostate cancer. This strategy appears particularly appealing for early glottic cancer, known to be of slow-growing nature and to rarely metastasize to lymph nodes [1]. While the implementation of SBRT schedules for laryngeal cancers has first been discouraged due to evidence of increased late toxicity, a recently published phase II trial demonstrated excellent oncological and functional outcomes for T1-T2 tumors [2], prompting the question whether SBRT is – or not the new way to irradiate early glottic cancer.

In the past decades, several options have been designed in order to preserve vocal function for early glottic cancer, such as transoral laser microsurgery (TLM), surgical strategies such as partial laryngectomy and radiation therapy (RT). Partial laryngectomy provides a local control reaching approximatively 90 % at 2 years for patients with T1 disease and ranging between 70 % to 90 % for patients with T2 disease [3,4]. Regarding functional outcomes, partial laryngectomy is associated with an improvement in voice parameters over time, demonstrated up to 1 year after surgery [5]. TLM involves removal of the gross tumor with minimal margins, and represents the preferred option for cTis or cT1a lesions due to both its excellent oncological and functional outcomes [6]. However, this strategy is associated with a higher risk of local recurrence in patients with T2 tumors and/or anterior commissural involvement. In this population of patients, local control rates ranges between 70 % to 85 %, while it usually reaches 95 % for T1 malignancies [4]. It is to be noted that functional outcomes following TLM is associated with the extent of resection, with larger resections being associated with poorer outcomes [7]. In patients with large tumors, the risk to develop hoarseness and breathiness tended to be higher with TLM compared with RT [8,9]. RT has been shown to provide also excellent local-control rates, reaching up to 94 % for T1 tumors and ranging between 70 % to 80 % for T2 tumors [4]. Several strategies have been developed both in terms of adaptation of dose and volume in the past years, in order to improve patients’ quality of life and mitigate toxicity. Regarding RT schedules, accelerated hypofractionated schedules (60–64.8 Gy/25-27fx or 56.25 Gy/25fx or 63 Gy/28fx) demonstrated their non-inferiority compared to normofractionated radiotherapy for the management of T1 and selected T2 (with no impaired cord mobility) [[10], [11], [12]]. Also, accelerated hypofractionated schedules were associated with an excellent toxicity profile, with few reports of grade 2 toxicity and no report of any grade 3 toxicity. Therefore, accelerated hypofractionated schedules represent a standard of care in this population of patients. In patients with T1 or limited T2 disease with no invasion of the ipsilateral supraglottic region, reduction of the target volume with single-cord irradiation resulted in a significant improvement in vocal function, together with a 5-year local control rate reaching 97 % [13]. In patients with large T2 tumors, the risk to develop nodal metastases appears to be higher and must discourage the implementation of target volume-reduction strategies. Indeed, within the RTOG-9512 trial, portal dimension was 6x6cm centered on the thyroid cartilage, thereby realizing an incidental irradiation of level II and III cervical nodes [14]. Therefore, from a radiobiological point of view, ultra-hypofractionated schedules hold the potential to increase tumor cell kill, and therefore translate into improved local control rates [15]. While SBRT might help preserve vocal function through the reduction in doses delivered to contralateral vocal cord and/or arytenoid, the optimal population to propose this strategy has been assessed within several prospective trials.

Schwartz *et al*. were the firsts to report outcomes of patients receiving SBRT for early stage glottic cancer, in a phase I study (Table 1) [16]. At one-year, disease-free survival reached 82 %, with 2 failures observed in cT2 tumors and 2 in situ/superficially invasive disease– all of these occurring in the high-dose volume, which is quite similar that outcomes with normofractionated and moderately hypofractionated RT [11,17]. No local failure was observed in patients receiving SBRT, at a dose of 42.5 Gy in 5 fractions, which was confirmed later on with a longer follow-up [18]. Later on, Kang *et al*. reported outcomes following either moderately hypofractionated RT or SBRT within a phase I trial [19]. Two accelerated RT regimens were tested: a moderately hypofractionated regimen performed at a dose of 59.5 Gy and 47.6 Gy in 17 fractions to the tumor and whole larynx, and a SBRT regimen at a dose of 55 Gy and 40.7 Gy in 11 fractions. Within the SBRT schedule, one patient experienced local relapse and subsequently died from disease. Recently, Sanguineti *et al.* reported excellent outcomes with 3-fractions SBRT for T1 glottic cancer, with a 100 % local control rate observed at 4 years [20]. Although these results seem encouraging, it to be noted that the number of patients included within these studies was very small. Also, large variations regarding the target volume exists between trials. In the study led by Yu *et al*., the high risk clinical target volume (CTV) consisted in the gross tumor with a 2 mm additional margins, while the whole larynx was included within the low-risk CTV [21]. Kang *et al*. also chose to include the entire larynx in the low-risk target volume [19]. With the accumulating knowledge on patterns of relapse following RT (including almost exclusively ipsilateral relapses) [22] and the demonstration of excellent tumor outcomes with single vocal cord irradiation [23], several teams took the leap towards the further reduction of target volumes for SBRT in early glottic cancer. In the study led by Schwartz *et al*., the CTV was defined as the gross tumor with a 2 mm expansion for T1 tumors. In case of T2 tumors, the CTV also included the ipsilateral vocal cord and paraglottic space. The arytenoid was included if the tumor was located within 2 mm or involving the anterior commissural space. In their phase II trial, Sher *et al*. recently demonstrated excellent oncological outcomes with SBRT performed on a restricted volume with only a internal grow tumor volume (iGTV) delineated and no additional margin to define a CTV [2]. A total dose of 42.5 Gy in 5 fractions was delivered, and the authors reported a cumulative incidence of local failure of only 8 % at 2 years, suggesting the safety of the approach on an oncological point of view, in selected patients [2].

**Table 1:** Studies assessing outcomes following sbrt for early stage glottic cancer.

Study	Design	Number of patients	Tumor stage	RT schedule	Technique	PTV margins	Follow-up	Toxicity	Oncological outcomes
Yu *et al*., 2018	Phase I	12 pts	T1a (66 %), T1b (17 %), T2 (17 %)	MHF: 55 Gy/11fx SBRT: 45 Gy/5fx	VMAT	3 mm	Dose level 1: 16.8 months Dose level 2: 9 months	No grade 3–4 toxicity	1 local relapse in dose level 2
Kang *et al*., 2019	Phase I	13 pts	T1a (70 %), T1b (15 %), T2 (15 %)	MHF: 59.5 Gy and 47.6 Gy/ 17 fx (daily) SBRT: 55 Gy and 40.7 Gy /11fx (every-other day or twice a week)	VMAT	3 mm	MHF arm: 37 months SBRT arm: 14.5 months	Acute: MHF: 28 % grade 2 laryngeal oedema SBRT: 33 % grade 2 laryngeal oedema Late: MHF: 0 % grade 2–3 laryngeal toxicity SBRT: 33 % grade 3 toxicity	MHF: PFS: 100 % SBRT: 1 local relapse at 4 months, died of disease
Schwartz *et al*., 2017 *et al*., 2019	Phase I	29 pts	Tis (3 %), T1a (52 %), T1b (21 %), T2 (24 %)	MHF: 50 Gy /15fx, 45 Gy/ 10fx SBRT: 42.5 Gy/5fx	CyberKnife Fiducial placement	3 mm	43.6 months	Acute: No grade 3–4 toxicity 45 Gy/10fx: 1 grade 4 laryngeal edema and grade 3 dysphagia 42.5 Gy/5fx: grade 3 laryngeal necrosis, dysphagia	Local control rate: 83 % No relapse in the SBRT arm
Sanguineti *et al*., 2024	Phase I-II	33 pts	T1a (70 %), T1b (30 %)	36 Gy/3fx (high-risk area), 30 Gy/3fx (low-risk areas) (every-other day)	VMAT	5 mm craniocaudal 3 mm elsewhere	51.5 months	Late: Grade 4 toxicity: 18.2 %	LCR (4 years): 100 %
Sher *et al*., 2025	Phase II	25 pts	Tis (4 %), T1a (64 %), T1b (20 %), T2 (12 %)	PTV volume < 10 cc and no active smoking: 42.5 Gy/5fx (twice a week) Other patients: 58.08 /16fx (daily)	NR	5 mm craniocaudal 3 mm elsewhere	3.7 years	No grade ≥ 3 toxicity	Local control rate: 92 %

Abbreviations: MHF: moderate hypofractionation, SBRT: stereotactic body radiotherapy, VMAT: volumetric modulated arctherapy, pts: patients, OS: overall survival, DFS: disease-free survival, Tis: in situ tumor, NR= not reported.

The dose received by the larynx has long been known to be associated with the onset of both acute and late toxicity, with doses over 70 Gy being associated with a 42 % risk to develop late laryngeal edema and cartilage necrosis [[24], [25], [26]]. Considering this, normofractionated RT schedules prescribed at a dose ranging between 66 to 70 Gy in 33–35 fractions are associated with a low risk to develop laryngeal necrosis, estimated to be of less than 2 % [13]. In contrast, considerable risk to develop laryngeal edema or necrosis have been reported in SBRT studies. Kang *et al*. reported a high rate of grade 3 laryngeal toxicity (33 %) with their SBRT schedule, with one report of a laryngeal ulcer and arytenoid cartilage necrosis requiring supraglottic laryngectomy [19]. The dose received by the ipsilateral arytenoid cartilage was suggested to be associated with severe toxicity, as the mean dose received by the cartilage raised up to 90.1 Gy within the SBRT schedule and was significantly higher than within the moderately hypofractionated arm (77.4 Gy). As this study suggested a potential interest in the implementation of dose-constraints on laryngeal sub-structures within SBRT trials, Sanguineti *et al*. also proposed to implement a Dmax to the cartilages at 30 Gy (in 3 fractions), with a priority given to CTV coverage on both high-risk and low-risk areas [20]. While this dose-constraint was respected for the arytenoid cartilage, it was not the case for both cricoid and thyroid cartilages, with Dmax exceeding dose-constraints in respectively 51.9 % and 96.3 % of the cases [27]. The study was stopped early due to the report of a 18.2 % rate of grade 4 toxicity consisting either mucosal necrosis (four patients) or cartilage necrosis (two patients), with a peak of prevalence at 20 months. All lesions healed with conservative treatment. The peak of toxicity was observed 19 days after SBRT, with a prevalence of grade 2 toxicity raising up to 84.8 %, completely resolving by nine weeks after SBRT. Other studies did not implement any constraints on laryngeal sub-structures and reported low rates of severe toxicity, suggesting that there is more to the picture than solely dosimetric considerations. Indeed, Sher *et al*. reported satisfactory toxicity outcomes within a dose-escalated phase I study, including both T1 and T2 disease [18]. Care was given to mitigate the dose delivered to the cartilage, but no dose-constraints was applied and doses incidentally delivered to ipsilateral arytenoid cartilage remained high, with a Dmax reaching 38 Gy and a mean dose of 31 Gy. One patient developed grade 4 laryngeal toxicity and subsequent local relapse. Retrospectively, this patient presented at diagnosis with T4 disease due to cricoid involvement, and was mis-staged. Only one patient in the 5 fractions schedule developed grade 3 dysphagia and laryngeal necrosis. In this study, PTV size was suggested to be associated with the onset of severe toxicity. Indeed, patients demonstrating severe toxicity had PTV size raising up to 17 cm^3^, while the median PTV size was only 5 cm^3^. In their phase II trial, only patients with PTV size less than 10 cm^3^ were deemed eligible for SBRT. No grade ≥ 3 toxicity was reported following a SBRT schedule delivering 42.5 Gy in 5 fractions [2]. Consequently, the onset of severe toxicity in the study led by Kang *et al*. can potentially be attributed to the inclusion of the whole larynx within the target volume, although the limited number of patients precludes any statistical analysis. Active smoking during and/or following SBRT has also been suggested to be associated with late severe toxicity. In the study led by Sanguineti *et al*., approximately 50 % of the patients kept smoking during and after SBRT. Consequently, active smoking with more than a pack per day was considered as an exclusion criteria in the recently study published by Sher *et al.* [2]. These findings highlight the need for a cautious patient selection when considering SBRT for early glottic cancer.

Preservation of vocal function represents a critical endpoint when considering the management of early glottic cancer. SBRT does not appear to be associated with a negative impact on vocal function. In the study led by Kang *et al*., speech parameters assessed with the HNCI questionnaire were lower in the SBRT arm at 1 months, with no difference observed with a longer follow-up. Sanguineti *et al*. reported a significant improvement in voice outcomes assessed with the Voice Handicap Index (VHI) at 6 months following SBRT [20]. Overall, voice function following SBRT appeared to be quite similar than what can be observed in patients receiving whole larynx normofractionated radiotherapy [28]. Sher *et al*. observed a continuous and significant improvement in vocal function, assessed with the VHI score, during the year following SBRT, in both their phase I and phase II trial [2,18]. The VHI score improved from 57 at baseline up to 7.5 at one year following SBRT, and remaining stable thereafter. Further analysis performed with electroglottography (EGG) support the fact that SBRT does not alter vocal function. Although an alteration is found immediately following radiotherapy in terms of motor control of the voice and hoarseness, these parameters have been shown to improve over time and return to baseline within months [19]. Although including the whole larynx within the CTV, Yu *et al*. reported an increase in maximum phonation time following SBRT, together with a non-significant decrease in jitter [21].

The place of alternative SBRT techniques such as magnetic resonance guided radiotherapy (MRgRT) is to date unanswered. Vocal cords have been shown to have few to no movement at rest, with vocal cord movement observed at less than 1.3 mm [29]. These findings have been confirmed in the phase I led by Schwartz *et al*., confirming the absence of vocal cord movement at different respiratory times [16]. Still, some studies performed treatment planning using 4-dimensional computed tomography (CT) and followed the internal target volume with the use of Synchrony motion tracking [2,18]. While vocal cords are mobile during deglutition, several studies instructed participants not to swallow during treatment delivery [18,20,21]. Little to no benefit is expected with adaptive radiotherapy in this disease setting. The benefit to implement strategies such as protontherapy for early glottic cancer SBRT has not been assessed. To date, only one study suggested a dosimetric improvement with the use of proton beam scanning (PBS) for the management of early glottic cancer, with a reduction in doses delivered to both inferior constrictor muscles and contralateral vocal cord [30].

The conclusions of this review, although systematic, are hampered by the short follow-up of the studies and the small number of patients included, which results in a form an unreliability regarding both oncological and toxicity outcomes. Also, these data need to be prospectively compared with other validated strategies such as partial laryngectomy or TLM.

SBRT for the management of early glottic tumors holds the potential to increase patient convenience and limit the costs of treatments. Fractionations such as 42.5 Gy in 5 fractions, performed every other day have been suggested to be safe, across few prospective trials. While some schedules demonstrated a higher risk of necrosis compared with moderately or normofractionated schedules, patient selection appears to be critical. Patients with limited tumor extent (10 cm^3^) and no active smoking probably represent the optimal population, although these findings need to be prospectively confirmed in larger cohorts. Also, patients to be considered for SBRT are those with little to no risk of nodal involvement, thereby limiting the implementation of this strategy in patients with T2 disease. Reducing PTV size through irradiation of the tumor-only with no additional CTV margin appears to provide the best functional outcomes while providing excellent tumor control rates. Last but not least, the place of SBRT needs to be prospectively compared to standard RT regimen and other treatment modalities (surgery and TLM) before being considered as a standard practice.

## Patient consent statement

The authors certify that no patient data was included in this work.

## CRediT authorship contribution statement

**Jennifer Le Guévelou:** Conceptualization, Methodology, Data curation, Writing – original draft, Visualization. **Audrey Larnaudie:** Writing – review & editing. **Pierre Blanchard:** Writing – review & editing, Supervision. **Yoann Pointreau:** Writing – review & editing, Supervision. **Joël Castelli:** Writing – review & editing, Supervision.

## Funding

None.

## Declaration of competing interest

The authors declare that they have no known competing financial interests or personal relationships that could have appeared to influence the work reported in this paper.
